# Investigation of Polymer-Assisted CO_2_ Flooding to Enhance Oil Recovery in Low-Permeability Reservoirs

**DOI:** 10.3390/polym15193886

**Published:** 2023-09-26

**Authors:** Xin Chen, Yiqiang Li, Xiaoguang Sun, Zheyu Liu, Jianbin Liu, Shun Liu

**Affiliations:** 1 College of Petroleum Engineering, Xi’an Shiyou University, Xi’an 710065, China; jianbin@xsyu.edu.cn; 2 Shaanxi Key Laboratory of Advanced Stimulation Technology for Oil & Gas Reservoirs, Xi’an 710065, China; 3 Ministry of Education Engineering Research Center of Development and Management for Low to Ultra-Low Permeability Oil & Gas Reservoirs in West China, Xi’an 710065, China; 4 State Key Laboratory of Oil and Gas Resources and Exploration and College of Petroleum Engineering, China University of Petroleum (Beijing), Beijing 102249, China; zheyu.liu@cup.edu.cn; 5 PetroChina Coalbed Methane Company Limited, Beijing 100028, China

**Keywords:** polymer-assisted CO_2_ flooding, CO_2_ responsive, injectivity, resistance increasing, profile control, EOR

## Abstract

CO_2_ flooding is a favorable technical means for the efficient development of low-permeability reservoirs, and it can also contribute to the realization of net-zero CO_2_ emissions. However, due to the unfavorable viscosity ratio and gravity overriding effect, CO_2_ channeling will inevitably occur, seriously affecting its storage and displacement effects. This paper conducts a systematic study on the application of polymer-assisted CO_2_ flooding in low-permeability reservoirs. Firstly, the polymer agent suitable for low-permeability reservoirs is optimized through the viscosity-increasing, rheological, and temperature- and salt-resistant properties of the solution. Then, the injectivity performance, resistance-increasing ability, and profile-improving effect of the polymer solution were evaluated through core experiments, and the optimum concentration was optimized. Finally, the enhanced oil recovery (EOR) effects of polymer-assisted and water-assisted CO_2_ flooding were compared. The results show that the temperature-resistant polymer surfactant (TRPS) has a certain viscosity-increasing performance, good temperature resistance performance, and can react with CO_2_ to increase the solution viscosity significantly. Meanwhile, TRPS has good injection performance and resistance-increasing effect. The resistance increasing factor (*η* and *η*′) of TRPS-assisted CO_2_ flooding increases with increased permeability, the concentration of TRPS solution, and injection rounds. Considering *η*′ and the profile improvement effect comprehensively, the application concentration of TRPS should be 1000 mg/L. The EOR effect of TRPS-assisted CO_2_ flooding is 8.21% higher than that of water-assisted CO_2_ flooding. The main effective period is in the first and second rounds, and the best injection round is three. The research content of this paper can provide data support for the field application of polymer-assisted CO_2_ flooding in low-permeability reservoirs.

## 1. Introduction

With increased international energy consumption and oil exploration and development intensification, low-permeability reservoirs have contributed to Chinese oil and gas resource production. Developing low-permeability oilfields is generally dominated by water injection, and gas flooding, especially CO_2_ flooding, is an efficient development technology for low-permeability reservoirs with excellent prospects [1,2]. CO_2_ flooding can play the role of high-efficiency oil displacement and geological storage simultaneously, in line with the policy of carbon peak and carbon neutrality, and has good application advantages. However, due to unfavorable mobility ratio and gravity overriding, CO_2_ will channel in the reservoir, significantly reducing the development effect [3,4,5]. Therefore, an economical and effective method for mitigating gas channeling is the key to further improving CO_2_ flooding in low-permeability reservoirs. Polymer flooding is the primary EOR technology in China [6], and it has been successfully applied in Daqing Oilfield, Xinjiang Oilfield, Dagang Oilfield, etc. [7,8]. Polymers can reduce the fluidity of injected fluids, reduce the permeability of advantageous channels through adsorption and retention, and ultimately expand the swept volume [9,10]. The resistance coefficient and residual resistance coefficient are usually used to evaluate the injectivity performance of polymer solutions, and having a suitable resistance coefficient and residual resistance coefficient is the prerequisite for the successful application of polymers [11]. Indoor research results show that polyacrylamide (HPAM) polymers can increase oil recovery by 10–12% of OOIP after water flooding [12]. Meanwhile, the molecular modification of the polymer can play an interface effect, stripping, dispersing, and carrying oil droplets [13]. In addition, polymer gels [14], polymer microspheres [15,16,17], polymeric surfactants [18], and other agents [19] can play a role in profile control and flooding, further improving oil recovery. A comparison of the EOR effects of different enhanced CO_2_ injection methods is shown in Appendix A Table A1. It can be found that as the system strength increases, the EOR gradually increases. However, due to the high viscosity characteristics of polymers, it must keep a good reservoir matching relationship while exerting the viscosity-increasing effect [20,21], so it is usually used in medium-high permeability reservoirs.

It is of excellent research significance to explore using polymers to assist CO_2_ flooding in low-permeability reservoirs to improve gas channeling and expand the swept volume. Dann et al. [22] compared the injection performance of polymer solutions in different low-permeability cores through core flooding experiments and pointed out that reservoir core characteristics other than permeability can dominate polymer performance. In addition, Ghosh et al. [23] conducted polymer injectivity experiments on two cores with a permeability of 15 mD but with significant differences in pore throat distribution. The results showed that the polymer could flow smoothly in the cores with a bimodal distribution of pore throats. But in another core with unimodal distribution, the flow is difficult. The above two studies show that the polymer solution has the potential to flow smoothly in the low-permeability reservoir, but it is necessary to meet the matching of the polymer and the reservoir. Marliere et al. [24] used cores with an effective permeability of 1–3 mD to carry out oil displacement experiments with polymers (molecular weight of 3 million Da and viscosity of 2 cP), which can increase oil recovery by 20% to 40% based on water flooding. Bennetzen et al. [25] proved by experiments that partially hydrolyzed polyacrylamide (HPAM, with a molecular weight of 8 million Da and a concentration of less than 5000 mg/L) solution injected into a 0.3 mD carbonate reservoir core at a rate of 0.5 mL/h will not produce core plugging. Mohammed Taha et al. [26] studied the application of low-salinity polymer flooding in high-temperature, high-salt, and low-permeability reservoirs. The polymers can maintain a viscosity of 2–3 cP (2500–4000 mg/L), with good injection and enhanced oil recovery effects. Leon et al. [27] reported a successful field pilot of the polymer in the low-permeability Palogrande-Cehu field. Considering the low permeability of the reservoir (6–150 mD), three polymers with different molecular weights (average concentration 1021 mg/L, viscosity 3.43 cP) were used, and the viscosity change was monitored. It is estimated that EOR efficiency can reach up to 8%, and the water cut of some wells is reduced by as much as 14%. The above studies show that the successful application of polymers in low-permeability reservoirs requires low viscosity and low injection velocity. For CO_2_ flooding in low-permeability reservoirs, a CO_2_ response mechanism based on the above studies [28,29,30] can be used to prevent gas channeling. Inject a low-viscosity polymer surfactant into the reservoir, which can react with CO_2_ to form worm-like micelles, significantly increasing the viscosity of the system [31,32]. However, due to the high viscosity of this type of micelles, it is mainly used for the plugging of fractures in low-permeability reservoirs [33,34,35]. Through the above literature review, two problems exist in the current research on polymer-assisted CO_2_ flooding in low-permeability reservoirs: (1) how to optimize the polymer agents with reservoir adaptability; and (2) how to apply the CO_2_ response characteristics in low-permeability reservoirs matrix.

Aiming at the above problems, the present paper studies a temperature-resistant CO_2_-responsive polymer surfactant (TRPS) to assist CO_2_ flooding to improve the oil recovery of low-permeability reservoirs. The advantages of it as an assisted agent were evaluated by comparing its static properties, such as viscosifying properties, rheological properties, and temperature and CO_2_ resistance with xanthan gum. Then, the injection performance, resistance-increasing performance, and profile improvement effect of TRPS were evaluated through core injectability experiments, and the optimal injection concentration was determined. Finally, the enhanced oil recovery effects of water-assisted CO_2_ flooding and TRPS-assisted CO_2_ flooding were compared. The research in this paper can provide the experimental basis and data support for agent selection, performance evaluation, and injection parameter optimization in the application process of polymer-assisted CO_2_ EOR of low-permeability reservoirs.

## 2. Material and Method

### 2.1. Material

Polymers: The injectivity performance of polymer solution in the low-permeability reservoirs can only be satisfied when the polymer molecular weight and solution viscosity are low enough. Low-molecular-weight polymers and polymeric surfactants can be used as two typical low-molecular and low-viscosity polymer systems. Here, xanthan gum (molecular weight is about 5 × 10^6^ Da) and a kind of CO_2_-responsive temperature-resistant polymeric surfactant (molecular weight is about 10^6^ Da) are selected as representatives of two polymer systems to conduct the following research. TRPS with certain interfacial activity and viscosity-increasing properties was obtained by grafting functional functional groups onto the main chain of polyacrylamide. Meanwhile, it has the CO_2_ corresponding characteristics and can significantly increase the viscosity of the solution in a CO_2_ environment.

Liquids: The inorganic salt purchased from Shanghai Aladdin Reagent Co. (Shanghai, China) was proportionally added to the deionized water (DI water) to prepare the simulated formation water, and the total salinity was 7000 mg/L. DI water was prepared by UPT-I-10T Ultra-pure Water Purifier from Chengdu Youpu Super Pure Technology Co. (Chengdu, China). The simulated oil is white oil purchased from, Shanghai Aladdin Co. (Shanghai, China), with a viscosity of 15.5 cP at 80 ℃ (target reservoir temperature).

**Cores:** Cylindrical Berea cores with a size of 3.8 × 30 cm were used for polymer-assisted CO_2_ flooding experiments, and the gas permeabilities are about 5 mD, 10 mD, and 20 mD, respectively. The effective permeability of each core was specifically tested before each experiment.

### 2.2. Polymers Static Properties

#### 2.2.1. Viscosity-Increasing Properties

The viscosity-increasing property of a polymer refers to the ability of a polymer to dissolve in water to increase the viscosity of the aqueous phase and is the most critical parameter for evaluating a polymer agent. Use DI water to prepare XG and TRPS mother liquor with a concentration of 5000 mg/L, and stir for 2 h with an electronic stirrer at 400 rpm. Then, the mother liquid could be diluted using simulated formation water to prepare the target polymer solution (100 mg/L, 300 mg/L, 500 mg/L, 700 mg/L, and 900 mg/L). The viscosities of the polymer solutions will be tested by a Brookfield viscometer after stirring for 1 h with an electronic stirrer at 200 rpm. The shear rate of the Brookfield viscometer is 7.4 1/s, and the test temperature is 80 °C.

Next, 100 mL of the XG and TRPS solutions with the target concentration were poured into the Warring agitator, respectively, sheared at 3500 rpm for 1 min, and then we tested the viscosity of the solution again and calculated the viscosity retention rate.

#### 2.2.2. Polymer Rheological Properties

The viscosity of polymer solution decreases with the increase in shear rate, which has a typical shear thinning property. Evaluating the viscosity of polymers at different shear rates is of great significance for testing their EOR efficiency. The rheological curves of XG and TRPS solutions at concentrations of 100 mg/L, 300 mg/L, 500 mg/L, and 1000 mg/L were tested using a Haake rheometer (HAAKE RS6000, Thermo Electron Karlsruhe GmbH, Karlsruhe, Germany), and the shear rate was 0.1 1/s to 1000 1/s, and the test temperature was 80 ℃.

#### 2.2.3. Polymer Reservoir Adaptability

After the polymer is injected into the reservoir, it needs to stay in the reservoir environment for a long time, and its performance under this condition determines its effect. The temperature resistance and CO_2_ resistance performance requirements of the polymer solution are evaluated by the viscosity retention rate to meet the application conditions of the oil reservoir. Put XG and TRPS solution with a concentration of 200 mg/L, 500 mg/L, 800 mg/L, 1000 mg/L, and 1500 mg/L in a high-temperature aging tank separately and aged in an oven at 80 °C for 30 days. Samples were taken at regular intervals (2, 4, 6, 8, 10, 15, 20, and 30 d) to test the viscosities using a Brookfield viscometer. In addition, injecting CO_2_ into piston containers with polymer solutions of 300 mg/L, 500 mg/L, and 800 mg/L until the pressure reaches 10 MPa. Then, seal the piston and place it at 80 ℃ for ten days for a certain period to test the viscosity of the solution.

### 2.3. The Flow Performance of TRPS in Low-Permeability Reservoirs

#### 2.3.1. The Injectivity Ability of TRPS

**Figure 1:**
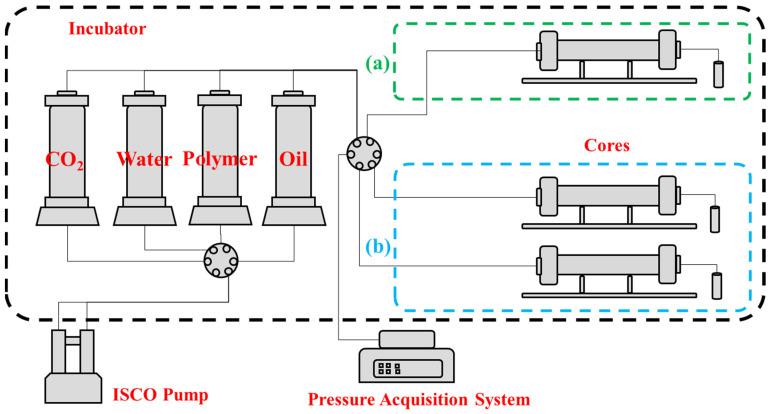
The flowchart of the flow performance of the TRPS solution. (**a**) Single core flooding flow chart, suitable for injectivity ability testing, resistance-increasing performance testing, and EOR efficiency evaluation. (**b**) Two-core parallel core flooding flow chart, suitable for profile control performance evaluation.

The injectivity performance of polymer solution is mainly quantitatively evaluated by Resistance Factor (R_F_) and Residual Resistance Factor (R_FF_). The specific experimental procedure is as follows: (1) After the core was vacuumed for 3 h, it was saturated with simulated water by self-priming for more than 4 h, and the porosity was calculated; (2) According to Figure 1a, the simulated formation water was injected with the ISCO pump at a constant rate, and the pressure difference between the two ends of the core was recorded as ΔP_1_ after the pressure became stable, and the core water permeability was calculated using Darcy law; (3) The ISCO pump was used to inject TRPS solution at a constant rate until the pressure is stable, and the pressure difference of the core was recorded as ΔP_2_; and (4) The ISCO pump was used to inject simulated formation water at a constant rate until the pressure was stable, and the pressure difference at both ends of the core was recorded as ΔP_3_. The injection rate is 0.3 mL/min, and the TRPS solution needs to be injected continuously for at least 2 PV. Finally, the FR = ΔP_2_/ΔP_1_ and the FRR = ΔP_3_/ΔP_1_ can be calculated. The specific experimental scheme is shown in Table 1.

In addition, a zeta potential analyzer (Zetasizer Nano ZS, Malvern Panalytical, malvern city, England) was used to test the hydrodynamic size of TRPS solutions with different concentrations, and the Poiseuille formula was used to calculate the mean pore throat size of the cores. The injectivity performance results of TRPS can be analyzed by comparing the above two sizes.

#### 2.3.2. Resistance-Increasing Performance of TRPS-Assisted CO_2_ Flooding

After clarifying the injectivity performance of TRPS, it is necessary to evaluate the resistance-increasing effect of TRPS in the presence of CO_2_. Evaluation is also carried out by RF and RFF. The specific experimental procedure is the same as that in Section 2.3.1, except that step (3) is to inject CO_2_ and TRPS solution into the core simultaneously using the ISCO pump. The specific experimental scheme is shown in Table 2.

Meanwhile, it is necessary to evaluate the resistance-increasing performance of the TRPS-assisted CO_2_ flooding process. Berea cores with a size of 3.8 cm × 20 cm and a permeability of 5 mD, 10 mD, and 20 mD were selected to evaluate the resistance-increasing effect of TRPS. The specific experimental process is as follows: (1) The core was vacuumed for 3 h and then saturated with water for more than 4 h by self-priming and calculating the porosity; (2) Connected the experiment flow chart in Figure 1a and carried out water flooding and gas flooding at a constant rate using the ISCO pump until the pressure is stable. Recorded the stable pressure P_0_ as the basic gas flooding pressure; (3) Injected TRPS solutions with concentrations of 200 mg/L, 500 mg/L, and 1000 mg/L at a constant rate, and recorded the stable pressure *P*_1_, respectively; (4) Carried out gas flooding again at a constant injection rate to record the pressure *P*_1_′ before gas channeling; and (5) Repeated Step (3) and Step (4) multiple times to obtain the pressures *P_i_* and *P_i_*′. TRPS resistance increase coefficient *η*_(i)_ = *P_i_*/*P*_0_ and resistance increase coefficient of gas flooding *η*′_(i)_ = *P*′*_i_*/*P*_0_.

#### 2.3.3. Profile Control Performance of TRPS

The 3.8 cm × 20 cm Berea cores with permeability of 5 mD and 20 mD were connected in parallel to simulate reservoir heterogeneity (permeability difference ratio is 4) to carry out the TRPS-assisted CO_2_ flooding experiment without oil. The specific experimental procedure is as follows: (1) After the core is evacuated for 3 h, it is saturated with simulated water by self-priming for more than 4 h, and the porosity is calculated; (2) The simulated formation water was injected at a constant rate by an ISCO pump, and the pressure at both ends of the core is recorded after the pressure is stable. The stable pressure difference is recorded as ΔP_1_, and the core water permeability is calculated by Darcy law; (3) According to Figure 1b, the experimental process was connected and simulated water and CO_2_ were co-injected using the ISCO pump. Recorded the liquid production conditions and injection pressure of the cores; (4) TRPS and CO_2_ were co-injected using the ISCO pump at a constant rate until the pressure is stable; and recorded the liquid production conditions and injection pressures of the two cores. The combined injection rate of liquid and gas is 0.6 mL/min (1:1 volume ratio), and if there is no CO_2_ injection, the TRPS injection rate is 0.6 mL/min. The specific experimental scheme is shown in Table 3.

### 2.4. EOR Efficiency of TRPS-Assisted CO_2_ Flooding

The oil displacement experiments were carried out using Berea cores (gas permeability is 10 mD and the water permeability is 5.19 mD). Comparing the oil recovery and pressure change law of water-assisted CO_2_ flooding and TRPS-assisted CO_2_ flooding, the advantages of TRPS-assisted CO_2_ flooding in improving oil recovery were clarified, and the best effect period was determined. The specific experimental procedure is as follows: (1) After the core is evacuated for 3 h, it is saturated with water by self-priming for more than 4 h, and the porosity is calculated; (2) The simulated formation water is injected at a constant rate by an ISCO pump, and the pressure at both ends of the core is recorded after the pressure is stable. The pressure difference is ΔP_1_, and the core water permeability is calculated by Darcy law; (3) According to the connection experiment process in Figure 1a, the ISCO pump is used to inject crude oil into the core at a constant rate until there is no water production, and the oil saturation is calculated; (4) Simulated formation water was injected with ISCO pump at a constant rate until the water cut reaches 90% and then switched to CO_2_ flooding until gas channeling without producing oil; (5) Injected TRPS solution (1000 mg/L) with ISCO pump at a constant rate of 0.2 PV and then switched to CO_2_ flooding until no oil is made again, this is a round of TRPS-assisted CO_2_ flooding process; (6) Repeated step (5) for a total of three times, that is, three rounds of TRPS-assisted CO_2_ flooding are completed. During the experiment, the fluid production and injection pressure of the core were recorded. The injection rate of liquid and gas was 0.3 mL/min. During the experiment, a back pressure of 10 MPa was applied at the outlet end of the core by a back pressure valve.

## 3. Result and Discussion

### 3.1. Static Performance Comparison between XG and TRPS

#### 3.1.1. Viscosity-Increasing Performance

**Figure 2:**
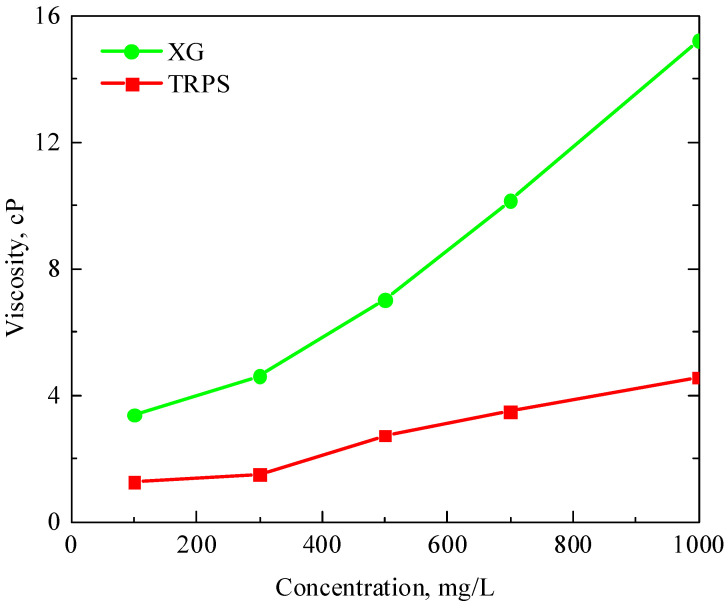
The viscosity-increasing ability of XG and TRPS.

Figure 2 shows the relationship curves of the viscosity of XG and TRPS polymer solutions with the change of solution concentration at 80 ℃. The results show that both polymers have certain viscosity-increasing properties in terms of demand in the field of oil and gas field development engineering, and the viscosity-increasing effect of XG is more significant. When the solution concentration exceeds 300 mg/L, the viscosity-increasing effect of the XG solution is significantly improved, much higher than that of TRPS at the same concentration. When the solution concentration was less than 1000 mg/L, the viscosity-increasing effect of TRPS gradually increased with the increase in the solution concentration, and the change was stable. When the concentration is 1000 mg/L, the viscosities of XG and TRPS solutions are 15.21 cP and 4.57 cP, respectively. Within this concentration range, XG and TRPS have the potential of low-permeability reservoir injectability [36], meeting the requirements of follow-up research. In addition, the viscosity of TRPS solutions with concentrations of 700 mg/L and 1000 mg/L is similar to that of XG solutions with concentrations of 100 mg/L and 300 mg/L, respectively. The performance of the two solutions at the same viscosity will be compared through the above two concentrations.

**Figure 3:**
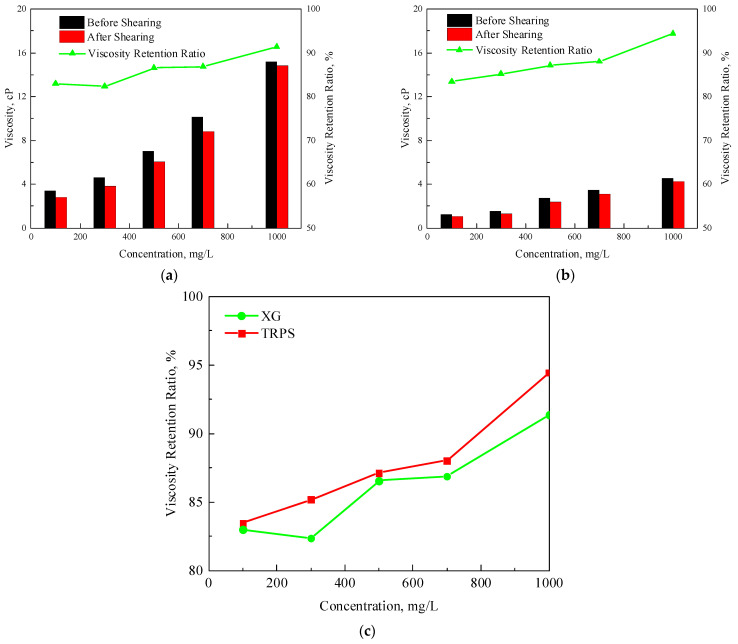
Viscosity and viscosity retention rate of polymer solution after shearing, (**a**) XG, (**b**) TRPS, (**c**) comparison of viscosity retention rate.

Figure 3a,b show that the viscosities and retention rates of XG and TRPS solutions increase with the increase in solution concentration. The relationship between the viscosity retention rate and the solution concentration of XG and TRPS solutions after shearing at 3500 rpm is compared, as shown in Figure 3c. The viscosity retention rates of the two polymer solutions after shearing are both above 80%. Whether the viscosity is the same or the concentration is the same, the viscosity retention rate of TRPS is greater than that of XG because the viscosity-increasing mechanism of XG and TRPS mainly includes: (1) the polymer molecules in water are entangled to form a structure; (2) The hydrophilic groups in the polymer chains are solvated in water, and the apparent molecular volume of the polymer increases. XG has a more considerable molecular weight, and the larger molecular aggregates formed are more easily damaged by shearing.

#### 3.1.2. Rheological Properties

**Figure 4:**
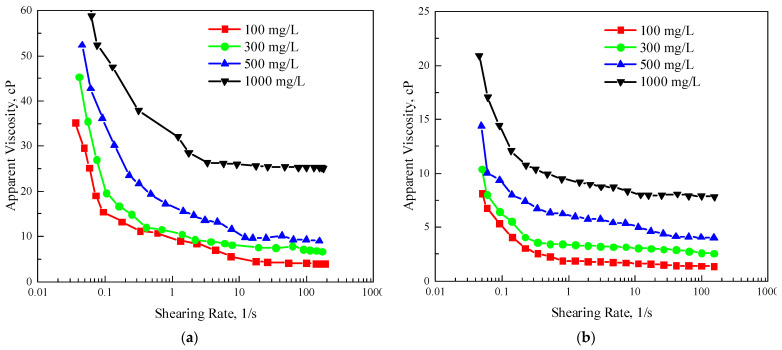
The rheological curve, (**a**) XG, (**b**) TRPS.

Figure 4 shows the rheological curves of XG and TRPS with four concentrations. Figure 4 shows that the rheological curves of XG and TRPS solutions are power-law curves, showing typical shear thinning characteristics. The shear rates corresponding to the rheological curves of XG and TRPS solutions reaching the second Newton zone are about 10 1/s and 5 1/s, respectively, and the molecular conformation transition has reached the limit value. Because in the practice of oil field production, the shear rate of the polymer system in the underground is about 1–10 1/s, combined with the rheological curve, it is easier for TRPS to reach the second Newton zone in this shear rate range. The adaptability to the reservoir shearing of TRPS is more robust. Meanwhile, comparing the rheological curves of TRPS (1000 mg/L) and XG (300 mg/L) solutions with the same viscosity, it can be found that the viscosity of XG solution is higher at low shear rate, and the viscosity of the two is similar in the second Newton zone. This is mainly because the molecular weight of XG is large, and the viscosity-increasing property of the molecular coil is stronger at a lower shear rate, and as the shear rate increases, the difference between the two molecular weights for the viscosity-increasing performance decreases.

#### 3.1.3. Temperature-Resistance and CO_2_-Resistance Performance

**Figure 5:**
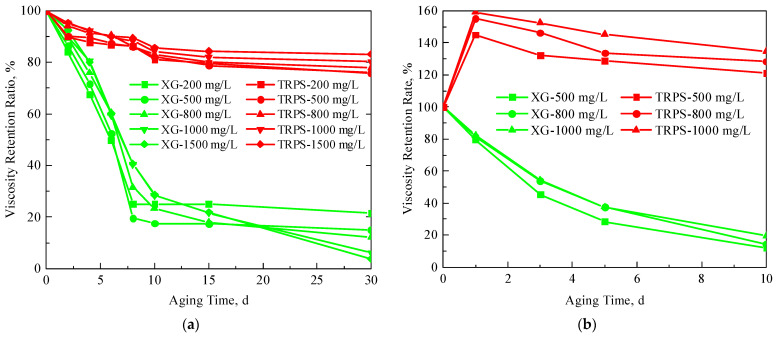
The viscosity retention ratio of XG and TRPS solutions, (**a**) high-temperature aging, (**b**) CO_2_ environment.

Figure 5 compares the viscosity retention curves of XG and TRPS solutions after aging in high-temperature and CO_2_ environments. Figure 5a shows that high temperature has a more significant effect on the viscosity of XG solution but has little impact on the viscosity of TRPS. The viscosity of the XG solution decreased rapidly with the increase in aging time, then reduced steadily after aging for ten days, and the viscosity retention rate after aging for 30 days was less than 20%. The viscosity of the TRPS solution decreased slowly with the increase in aging time, and the final viscosity retention rate was about 80%. This is because high-temperature conditions will gradually untangle the initially entangled polymer coils, and long-term aging will cause further breakage of the stretched molecular bonds, resulting in a significant decrease in the viscosity of the solution. However, TRPS has a temperature-resistant monomer and a short molecular coil, showing good temperature resistance.

**Figure 6:**
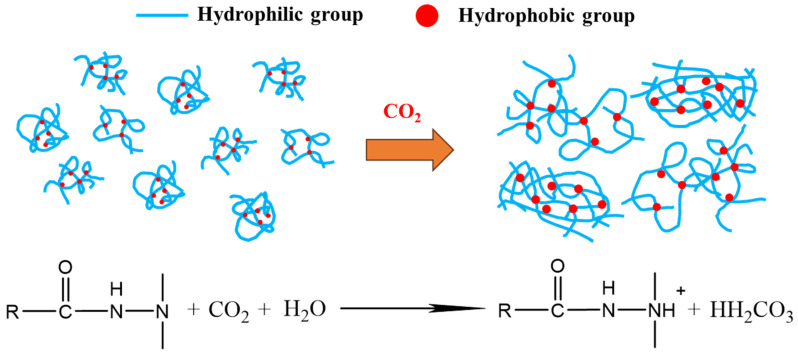
Schematic diagram of the mechanism of viscosity increase in response to TRPS and CO_2._

Figure 5b shows that the presence of CO_2_ significantly reduces the viscosity of the XG solution, and the viscosity retention rate drops to about 20% after aging for ten days. However, after aging in a CO_2_ environment, the viscosity retention rate of TRPS solution increased first (up to more than 150%), then decreased slowly, and finally remained at about 120%. CO_2_ will increase H^+^ in the polymer solution and destroy the biomolecular chains in the XG solution, thereby losing the viscosity-increasing effect [37]. The TRPS molecular chain contains tertiary amine groups with CO_2_ response characteristics. The primary tertiary amine group can undergo an acid-base neutralization reaction with dissolved CO_2_ in aqueous solution to form a bicarbonate structure. The tertiary amine group is protonated to form a quaternary salt cation structure, and the molecular chain is hydrophilic enhanced. Therefore, the presence of CO_2_ leads to larger TRPS molecular aggregates and an increase in the viscosity of the system [38,39]. The specific reaction mechanism is shown in Figure 6.

In addition, it can be seen from Figure 5 that regardless of the concentration of the TRPS solution, the viscosity retention rate after high-temperature aging and CO_2_ environment aging is higher than that of XG. This also shows that under the same viscosity, the temperature resistance and CO_2_ resistance of the TRPS solution are better than XG.

### 3.2. Injectivity of TRPS

**Figure 7:**
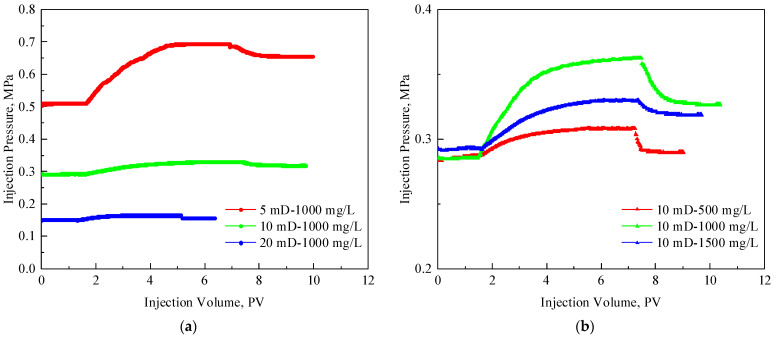
TRPS injection pressure curve. (**a**) The TRPS concentration is 1000 mg/L, and the core permeability is 5 mD, 10 mD, and 20 mD. (**b**) The core permeability is 10 mD, and the TRPS concentration is 500 mg/L, 1000 mg/L, and 1500 mg/L.

Figure 7 shows the TRPS injection pressure curves under three concentrations and three core permeabilities. The injection pressure increased significantly during the TRPS flooding, and it decreased slightly and then remained stable in the subsequent water displacement stage. It also showed a certain resistance-increasing ability under the condition of no CO_2_. R_F_ and R_FF_ increase with the increase in TRPS concentration and the decrease in core permeability. When the concentration of TRPS is fixed at 1000 mg/L, R_F_ and R_FF_ in 5 mD core are 1.36 and 1.28, respectively, indicating good injectivity. However, the R_F_ and R_FF_ of 1000 mg/L TRPS solution in 20 mD cores are both less than 1.1, which means that although its resistance-increasing effect is poor, its injectivity is good. R_F_ and R_FF_ of 500 mg/L, 1000 mg/L, and 1500 mg/L TRPS solutions in 10 mD cores are, respectively, distributed between 1.08–1.27 and 1.01–1.15, indicating that the concentration of TRPS solution will not significantly influence its injectivity. When the core permeability is greater than 5 mD, and the TRPS solution concentration is less than 1500 mg/L, R_FF_ is close to 1, indicating that the TRPS solution has less adsorption and retention in the formation, less damage to the formation, and has good injection performance.

**Figure 8:**
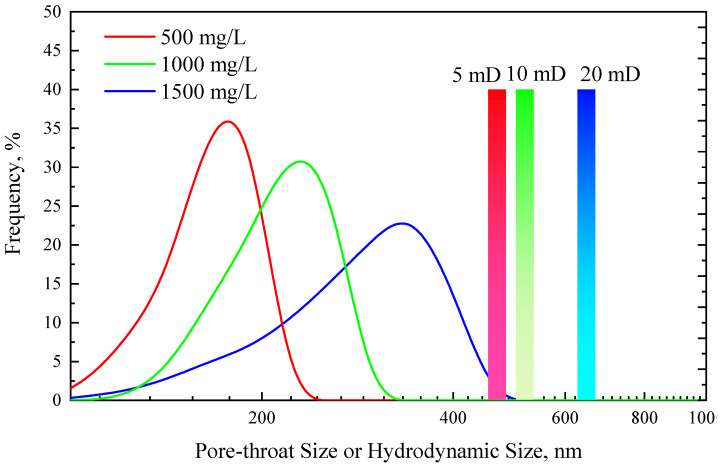
Comparison of the pore throat sizes of cores and the hydrodynamic sizes of TRPS solutions.

Figure 8 compares the hydrodynamic size of the TRPS solution and the mean pore throat size of the core. It can be found that as the concentration of TRPS solution increases, its hydrodynamic size gradually increases from 150 nm to 350 nm, and no obvious intermolecular association occurs. As the core permeability increases, the mean pore throat size increases from 470 nm to 650 nm. The mean pore throat size of TRPS is smaller than that of the core, ensuring its good injectivity performance. Meanwhile, TRPS molecules can effectively plug pore throats through the 1/2 and 1/3 bridging theory. This can explain why TRPS in Figure 7 can effectively increase the injection pressure while ensuring a low resistance coefficient.

### 3.3. Resistance Increasing Ablity of TRPS

**Figure 9:**
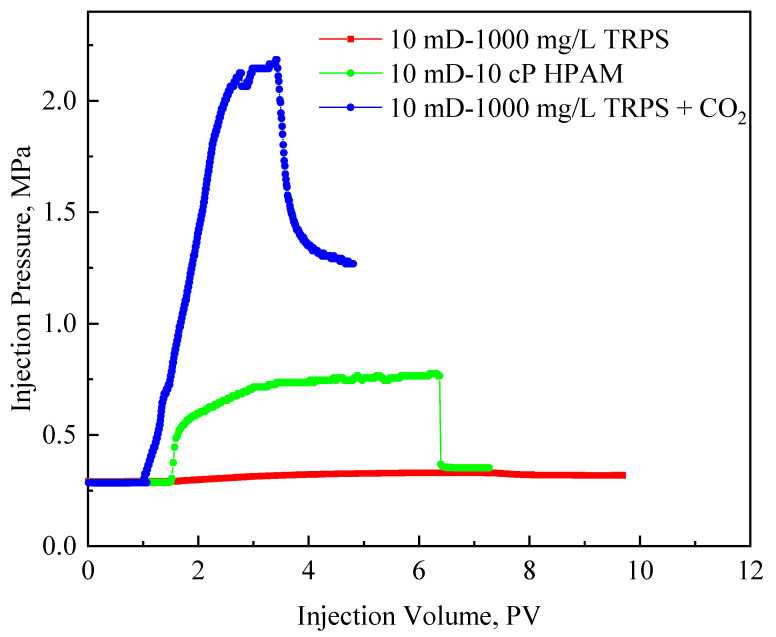
The injection pressure curves of TRPS injection, HPAM injection, and co-injection of TRPS and CO_2._

Figure 9 shows the injection pressure curves of the TRPS injection, HPAM injection, and co-injection of TRPS and CO_2_. The injection pressure rises rapidly after HPAM injection, which is much higher than that of TRPS injection, showing that even if the viscosity of the polymer solution is the same, the molecular weight of the polymer also affects its injection performance and resistance-increasing performance. The R_F_ of HPAM injection is 7.67, which is not significant in value, but the injection pressure rises above 2.0 MPa, and there will be a problem with too high injection pressure during the oilfield application. Meanwhile, the R_FF_ of HPAM injection is 4.45, indicating that HPAM has a large amount of retention and adsorption in the low-permeability layer, which is not conducive to the subsequent development of the formation. However, the R_F_ and R_FF_ of TRPS injected are 1.13 and 1.09, respectively, and the resistance-increasing effect is poor. The injection pressure during the co-injection of TRPS and CO_2_ increases significantly because the more complex molecular aggregates formed after the mixing of TRPS and CO_2_ significantly increase its flow resistance (Figure 6). At this time, R_F_ and R_FF_ rose to 2.67 and 1.23, respectively, and the effect of increasing resistance was improved. Figure 9 shows that it is difficult for conventional polymers (relatively high molecular) to achieve the coordination of injectivity and resistance-increasing in low-permeability reservoirs, while the response of TRPS and CO_2_ allows it to be injected into the reservoir smoothly and achieve resistance increase inside the reservoir effect.

**Figure 10:**
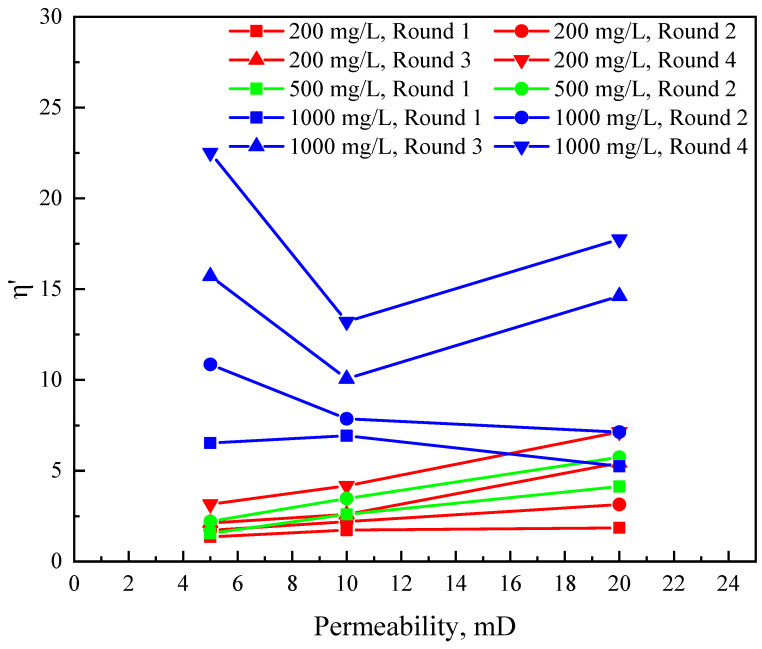
The resistance-increasing coefficient chart.

The injection pressures of multiple rounds of TRPS-assisted CO_2_ flooding with three kinds of permeability and three kinds of concentrations are collected and the resistance increase coefficients *η* and *η*′ of each round are shown in Table 4. Table 4 shows that *η* is greater than *η*′ in the same round because the resistance of the injected liquid must be greater than the resistance of the injected CO_2_. Under the same permeability conditions, *η* and *η*′ increased significantly after increasing the concentration of TRPS. When the concentration is 1000 mg/L, *η* can reach more than three times that when the concentration is 200 mg/L. *η*′ can reflect the resistance-increasing effect after the reaction of TRPS and CO_2_, so the relationship between *η*′ of the above rounds and the change of core permeability is plotted into a resistance-increasing coefficient chart, as shown in Figure 10.

When the concentration of TRPS is lower than 1000 mg/L, the increase in core permeability and alternate rounds will increase *η*′. This is because although the increase in permeability will reduce the injection pressure of TRPS-assisted CO_2_ flooding, the gas channeling pressure of CO_2_ flooding will also decrease, eventually increasing *η*′. The molecular aggregates produced by the reaction of TRPS and CO_2_ will be adsorbed and retained in the core, so *η*′ increases with the increase in injection rounds. When the concentration of TRPS is 1000 mg/L, the relationship curves of *η*′ change abnormally. This also shows once again the matching between TRPS and the reservoir (the low-permeability reservoir will not have unusually high pressure caused by the poor injectivity of molecular aggregates produced after the reaction of TRPS and CO_2_) and the cumulative effect of multiple rounds of resistance-increasing performance. When the permeability is 5 mD, the molecular aggregates formed by the reaction of TRPS and CO_2_ are poorly compatible with the core, resulting in an abnormal increase in injection pressure and a significant increase in *η*′. Then, the pore throats of the 10 mD and 20 mD cores became more prominent, and the flow capacity increased, which relieved the contradiction between the poor matching between TRPS and the core, and the change law of *η*′ was normal. In addition, the irregularity of *η*′ of the TRPS solution with a concentration of 1000 mg/L increases with the increase in injection rounds, mainly in the core with a permeability of 5 mD. This is due to the increase in retention of TRPS in the core due to the decrease in permeability.

### 3.4. Profle Control Effect of TRPS

**Figure 11:**
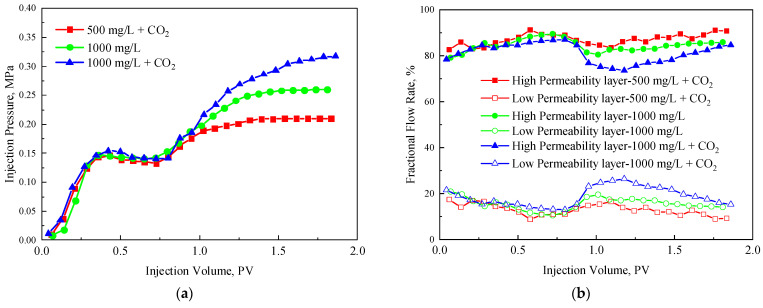
TRPS-assisted CO_2_ parallel flooding characteristic curves. (**a**) Injection pressure curves, (**b**) fractional flow rate curve.

Figure 11 shows the injection pressure and fractional flow rate curves of the TRPS-assisted CO_2_ flooding in a two-core parallel model. Figure 11 shows that the injection pressure of 1000 mg/L TRPS-assisted CO_2_ flooding is higher than that of TRPS flooding separately, which also leads to a more obvious reduction in the fractional flow rate of the increased permeability layer. The injection pressure of TRPS-assisted CO_2_ flooding with a concentration of 500 mg/L is lower than that of TRPS separately (1000 mg/L), showing that TRPS needs to reach a certain concentration before fully reacting with CO_2_. Combined with the resistance-increasing effect of TRPS, it can be determined that the optimal concentration of TRPS should be 1000 mg/L for reservoirs with a permeability above 5 mD and 500 mg/L for reservoirs below 5 mD.

### 3.5. EOR Effects of TRPS

**Figure 12:**
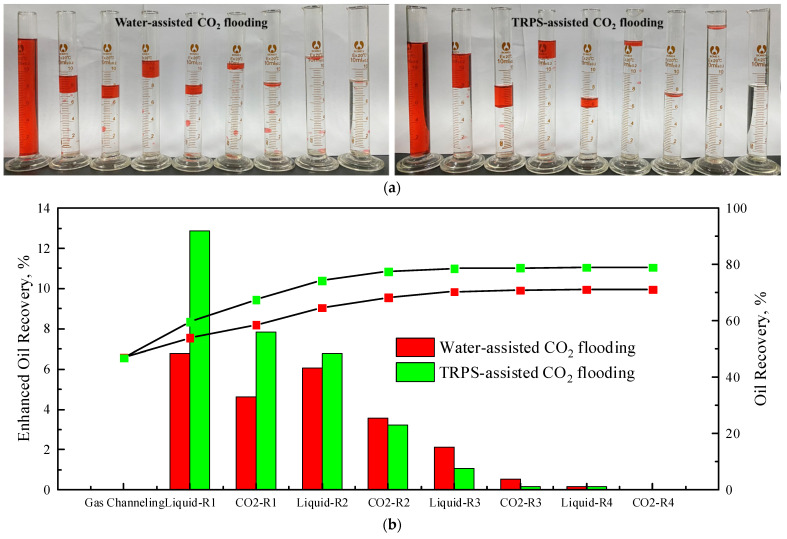
Experimental photos and data analysis of water-assisted CO_2_ flooding and TRPS-assisted CO_2_ flooding. (**a**) Liquid production photos, (**b**) EOR curves of each round.

Figure 12 shows the produced fluids and oil recovery curves of each stage for water-assisted CO_2_ flooding and TRPS-assisted CO_2_ flooding. CO_2_ can dissolve in crude oil and cause it to expand, reducing its viscosity [40,41], and has a higher recovery rate than air and N_2_. The oil recovery after CO_2_ flooding to gas channeling is about 46%, and liquid-assisted CO_2_ flooding can effectively control the gas channeling and further improve the oil recovery. Whether it is water or TRPS solution, the enhanced oil recovery of each round of the liquid injection stage is higher than that of the CO_2_ injection stage of the same round. TRPS-assisted CO_2_ flooding enhances oil recovery mainly in rounds 1 and 2, and the EOR effects are 20.71% and 10.00%, respectively. This also led to poorer EOR effects in subsequent rounds, in which the EOR in the third round was lower than that of water-assisted CO_2_ flooding. The main effect period of water-assisted CO_2_ flooding is in the first three rounds, but the overall EOR effect is 8.21% lower than that of TRPS-assisted CO_2_ flooding. The ultimate recovery of TRPS-assisted CO_2_ flooding and water-assisted CO_2_ flooding can reach 78.93% and 71.07%, respectively. 

Appendix A Table A1 compares the EOR effects of different CO_2_ enhancement injections. It can be found that compared with the ultrasonic physical method and another polymer-assisted CO_2_ displacement, the TRPS used in this paper has a significantly better EOR effect. In the literature review, only two scenarios obtained higher EOR than this paper (the bolded items in Appendix A Table A1). The first scenario is for heavy oil reservoirs, where CO_2_ or WAG recovery is very low due to the extremely unfavorable mobility ratio, so the effect of polymer-assisted CO_2_ displacement is remarkable. The second scenario is gel particle-assisted CO_2_ flooding, and the research target is parallel cores. Because the profile improvement effect of gel particles is stronger than that of polymers, its EOR effect in heterogeneous reservoirs is remarkable. Therefore, the TRPS-assisted CO_2_ flooding in this paper has a considerable EOR effect of 32.93% compared with CO_2_ flooding and 8.21% compared with water-assisted CO_2_ flooding.

**Figure 13:**
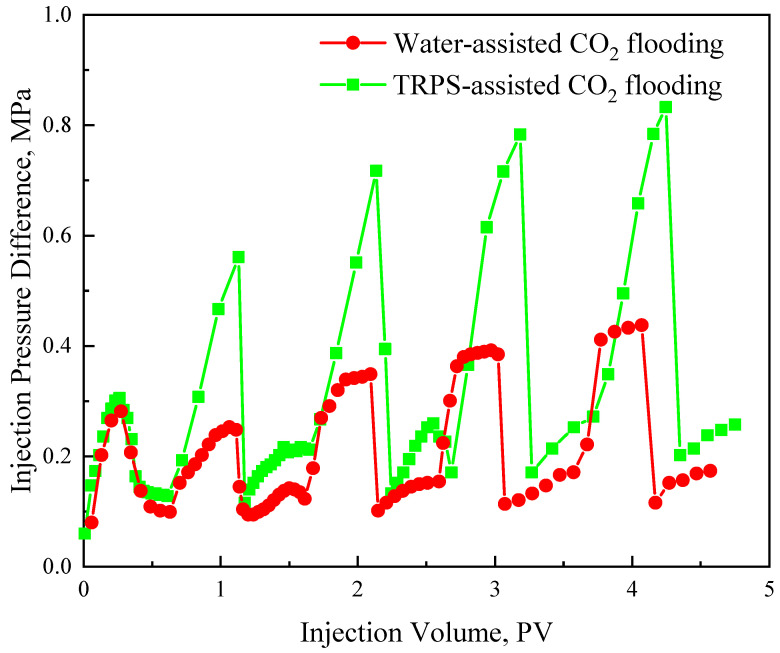
The injection pressure difference curves of water-assisted CO_2_ flooding and TRPS-assisted CO_2_ flooding.

Figure 13 is the pressure difference comparison curve of water-assisted CO_2_ flooding and TRPS-assisted CO_2_ flooding. The increase in injection pressure during the liquid injection is more significant than that of the gas injection, which can also explain the better EOR effect of each round of liquid injection in Figure 12. For water-assisted CO_2_ flooding, the gas-injection pressure in each round is close to the initial gas channeling pressure, indicating that gas–water alternation can increase the flow resistance of two-phase flow to a certain extent, but the gas channeling will quickly occur and form a dominant channel again. However, the CO_2_ injection pressure of each round of TRPS-assisted CO_2_ flooding is significantly higher than that of water-assisted CO_2_ flooding because the molecular aggregates produced by TRPS can effectively increase the flow resistance and adsorption time in the channeling channel. Figure 13 shows that, whether water or TRPS solution, the injection pressure of each round of the water injection stage and the end of the CO_2_ injection stage is significantly higher than that of the previous round, which has a significant cumulative effect. The higher injection pressure is the main reason for the quicker effect of TRPS-assisted CO_2_ flooding and better EOR effect. The injection pressure of liquid-assisted CO_2_ flooding will continue to increase with the injection round, but the EOR will drop significantly after the third round, so the optimal development round should be three. In addition, the increased injection pressure difference of liquid-assisted CO_2_ displacement can not only expand the swept volume [42], but also effectively promote the dissolution of CO_2_ into crude oil and reduce its viscosity. The high-pressure CO_2_ environment will reduce the viscosity of the TRPS solution, but the viscosity of crude oil will be reduced to a greater extent, so TRPS-assisted CO_2_ flooding has good EOR potential.

## 4. Conclusions

This paper investigated the improvement of CO_2_ channeling in low-permeability reservoirs by polymer solutions. Through the experimental evaluation of the static properties, flow properties, resistance-increasing effect, and EOR effect of the polymer solution, the polymer surfactant (TRPS) with CO_2_ response was selected as the assisted agent, which has a good application effect. The specific conclusions are as follows:

(1) TRPS has a certain viscosity-increasing property, and the viscosity of the solution is 4.57 cP when the concentration is 1000 mg/L. It has good temperature resistance, and its viscosity retention rate is above 80% after aging at 80 °C for 30 days. In addition, TRPS can react with CO_2_ to increase the size of molecular aggregates significantly, and the viscosity retention rate is about 120% after aging in a CO_2_ environment for 10 days.
(2) The R_F_ and R_FF_ of the TRPS solution with a concentration of 1000 mg/L in 5 mD cores are 1.36 and 1.28, respectively, showing good injectivity performance. Increasing the concentration of TRPS to 1500 mg/L had little effect on its injectivity performance.
(3) The injection pressure of TRPS and CO_2_ co-injection is between the injection of HPAM with the same viscosity and the injection of TRPS solution alone, which has good flow performance and resistance-increasing effect. The *η* and *η*′ of TRPS-assisted CO_2_ flooding increase with increased permeability, concentration of TRPS solution, and injection rounds. When the permeability is 5 mD, the base pressure of gas channeling is high, which will reduce the matching system between TRPS and the reservoir, thus affecting the change law of *η*′.
(4) The effect of TRPS solution on profile improvement: 1000 mg/L TPRS + CO_2_ > 1000 mg/L TRPS > 500 mg/L TRPS + CO_2_, considering the *η*′ and profile improvement effect, the application concentration of TRPS should be 1000 mg/L.
(5) The EOR effect of TPRS-assisted CO_2_ flooding is 8.21% higher than that of water-assisted CO_2_ flooding. The EOR effect of TRPS-assisted CO_2_ displacement is mainly reflected in the first to second rounds, while the EOR effect of water-assisted CO_2_ displacement is primarily reflected in the first to third rounds. The injection pressure of liquid-assisted CO_2_ flooding has a cumulative impact of multiple rounds, so the optimal injection round is 3.

## Figures and Tables

**Table 1:** The experimental scheme of TRPS injectivity properties.

Polymer Concentration, mg/L	Gas Permeability, mD	Water Permeability, mD	Core Volume, cm^3^	Pore Volume, cm^3^	Porosity, %
1000	5	2.99	226.7	24.32	10.73
500	10	5.36	226.7	34.28	15.12
1000	10	5.23	226.7	35.82	15.8
2000	10	5.38	226.71	33.56	14.80
1000	20	10.12	226.71	43.80	19.32

**Table 2:** Scheme of the evaluation of the resistance-increasing performance of TRPS.

Solution	Viscosity, cP	Injection CO_2_	Gas Permeability, mD	Water Permeability, mD	Porosity, %
TRPS	9.3	Yes	10	5.20	15.51
HPAM	10.1	No	10	5.36	14.98
TRPS	9.3	No	10	5.32	15.11

**Table 3:** TRPS profile control effect experimental scheme.

Solution	Concentration, mg/L	Injection CO_2_	Gas Permeability, mD	Water Permeability, mD
TRPS	500	Yes	5/20	3.12/9.98
TRPS	1000	No	5/20	3.03/10.20
TRPS	1000	Yes	5/20	3.21/10.15

**Table 4:** *η* and *η*′ of each round of TRPS-assisted CO_2_ flooding.

Parameters			Round 1		Round 2		Round 3		Round 4	
Permeability, mD	Concentration, mg/L	Gas Channeling Pressure, MPa	*η*	*η*′	*η*	*η*′	*η*	*η*′	*η*	*η*′
5	200	0.25	1.96	1.36	3.08	1.72	3.92	2.12	5.60	3.16
	500	0.33	2.70	1.55	6.18	2.21	/	/	/	/
	1000	0.31	6.74	6.53	11.2	10.8	16.1	15.7	22.9	22.5
10	200	0.13	2.20	1.73	3.23	2.20	4.96	2.60	9.37	4.17
	500	0.15	5.00	2.60	9.53	3.47	/	/	/	/
	1000	0.14	8.43	6.93	12.4	7.86	20.0	10.0	28.0	13.2
20	200	0.07	2.57	1.86	4.43	3.14	7.43	5.43	10.4	7.14
	500	0.08	7.50	4.13	14.1	5.75	/	/	/	/
	1000	0.08	8.50	5.25	18.0	7.13	29.1	14.6	42.3	17.7

## Data Availability

Not applicable.

## Appendix A

**Table A1:** EOR comparison of CO_2_ flooding with different strengthening methods.

Number	Literature	Contents	Agent	Models	EOR
0	This paper	Polymer-assisted CO_2_ flooding	CO_2_-responsive polymer, TRPS	Cores, 5–20 mD	Based on CO_2_ flooding is 32.93%; based on water-assisted CO_2_ flooding is 8.21%
1	Chaturvedi [43]	Polymer-enhanced carbonated water injection	PAM	Sandpack, ~780 mD	Based on water flooding is 16.00%
2	Zhao [44]	CO_2_ foam	AOS	Microfludic	Based on water flooding is 13.8–22.4%
3	Hossein [45]	Ultrasound-assisted CO_2_ flooding	/	Sandpack	Based on CO_2_ flooding is less than 15%
4	Li [42]	Polymer-assisted CO_2_ flooding	Polymer	Simulation, 1–2000 mD	Based on water flooding ranges from 20–30%
5	Luo [33]	Polymer-assisted CO_2_ flooding	Thermo- and CO_2_-triggered copolymer	Core	Based on water flooding is 21–23%
6	Yang [46]	Polymer-assisted CO_2_ flooding in heavy oil reservoir	Polymer	Simulation, 500 mD	**Based on WAG is 57%**
7	Gandomkar [47]	Polymer thickening CO_2_ flooding	Polydimethylsiloxane (PDMS)	Core, 6–8 mD	Based on CO_2_ flooding is 6–15%
8	Manan [48]	Polymer/surfactant/nano-particles assisted CO_2_ flooding	AOS, NPs (TiO_2_, CuO, SiO_2_, and Al_2_O_3_)	Sandpack	Based on water flooding is about 5.1–15.6%
9	Zaberi [49]	Polymer-assisted CO_2_ flooding	Polyfluoroacrylate (PFA)	Berea sandstone, ~31 mD	Based on CO_2_ flooding is 16%
10	Liu [5]	Microgel alternate CO_2_	Microgel	Core, 2800 mD/780 mD/360 mD	**Based on WAG is 12.4%**

## References

[1] Kang W.-L., Zhou B.-B., Issakhov M., Gabdullin M. (2022). Advances in enhanced oil recovery technologies for low permeability reservoirs. Pet. Sci..

[2] Chen Z., Su Y.-L., Li L., Meng F.-K., Zhou X.-M. (2022). Characteristics and mechanisms of supercritical CO_2_ flooding under different factors in low-permeability reservoirs. Pet. Sci..

[3] Zhao F., Wang P., Huang S., Hao H., Zhang M., Lu G. (2020). Performance and applicable limits of multi-stage gas channeling control system for CO_2_ flooding in ultra-low permeability reservoirs. J. Pet. Sci. Eng..

[4] Liu F., Yue P., Wang Q., Yu G., Zhou J., Wang X., Fang Q., Li X. (2022). Experimental Study of Oil Displacement and Gas Channeling during CO_2_ Flooding in Ultra—Low Permeability Oil Reservoir. Energies.

[5] Liu Z., Zhang J., Li X., Xu C., Chen X., Zhang B., Zhao G., Zhang H., Li Y. (2022). Conformance control by a microgel in a multi-layered heterogeneous reservoir during CO_2_ enhanced oil recovery process. Chin. J. Chem. Eng..

[6] Youyi Z., Qingfeng H., Guoqing J., Desheng M.A., Zhe W.A.N.G. (2013). Current development and application of chemical combination flooding technique. Petroleum Explor. Develop..

[7] Wang D., Zhang Z., Cheng J., Yang J., Gao S., Li L. (1997). Pilot Tests of Alkaline/Surfactant/Polymer Flooding in Daqing Oil Field. SPE Reserv. Eng..

[8] Yupu W., He L. Commercial Success of Polymer Flooding in Daqing Oilfield—Lessons Learned. Proceedings of the SPE Asia Pacific Oil and Gas Conference and Exhibition.

[9] Mishra S., Bera A., Mandal A. (2014). Effect of Polymer Adsorption on Permeability Reduction in Enhanced Oil Recovery. J. Pet. Eng..

[10] Kumar S., Mandal A. (2017). Rheological properties and performance evaluation of synthesized anionic polymeric surfactant for its application in enhanced oil recovery. Polymer.

[11] Verma J., Mandal A. (2021). Potential effective criteria for selection of polymer in enhanced oil recovery. Pet. Sci. Technol..

[12] Guangzhi L.I.A.O., Qiang W.A.N.G., Hongzhuang W.A.N.G., Weidong L., Zhengmao W. (2017). Chemical flooding development status and prospect. Acta Petrolei Sin..

[13] Li X., Zhang F., Liu G. (2021). Review on polymer flooding technology. IOP Conference Series: Earth and Environmental Science.

[14] Liu J., Zhong L., Wang C., Li S., Yuan X., Liu Y., Meng X., Zou J., Wang Q. (2020). Investigation of a high temperature gel system for application in saline oil and gas reservoirs for profile modification. J. Pet. Sci. Eng..

[15] Chen X., Li Y., Liu Z., Zhang J., Trivedi J., Li X. (2023). Experimental and theoretical investigation of the migration and plugging of the particle in porous media based on elastic properties. Fuel.

[16] Chen X., Li Y., Liu Z., Zhang J., Chen C., Ma M. (2020). Investigation on matching relationship and plugging mechanism of self-adaptive micro-gel (SMG) as a profile control and oil displacement agent. Powder Technol..

[17] Chen X., Li Y., Liu Z., Li X., Zhang J., Zhang H. (2020). Core- and pore-scale investigation on the migration and plugging of polymer microspheres in a heterogeneous porous media. J. Pet. Sci. Eng..

[18] Chen X., Li Y.-Q., Liu Z.-Y., Trivedi J., Gao W.-B., Sui M.-Y. (2023). Experimental investigation on the enhanced oil recovery efficiency of polymeric surfactant: Matching relationship with core and emulsification ability. Pet. Sci..

[19] Zhong L., Liu J., Yuan X., Wang C., Teng L., Zhang S., Wu F., Shen W., Jiang C. (2020). Subsurface Sludge Sequestration in Cyclic Steam Stimulated Heavy-Oil Reservoir in Liaohe Oil Field. SPE J..

[20] Hu J., Li A., Memon A. (2020). Experimental Investigation of Polymer Enhanced Oil Recovery under Different Injection Modes. ACS Omega.

[21] Liang X., Shi L., Cheng L., Wang X., Ye Z. (2021). Optimization of polymer mobility control for enhanced heavy oil recovery: Based on response surface method. J. Pet. Sci. Eng..

[22] Dann M.W., Burnett D.B., Hall L.M. Polymer performance in low permeability reservoirs. Proceedings of the SPE International Conference on Oilfield Chemistry.

[23] Ghosh P., Sharma H., Mohanty K.K. (2018). ASP flooding in tight carbonate rocks. Fuel.

[24] Marliere C., Wartenberg N., Fleury M., Tabary R., Dalmazzone C., Delamaide E. Oil Recovery in Low Permeability Sandstone Reservoirs Using Surfactant-Polymer Flooding. Proceedings of the SPE Latin America and Caribbean Petroleum Engineering Conference.

[25] Bennetzen M.V., Gilani S.F., Mogensen K., Ghozali M., Bounoua N. Successful polymer flooding of low-permeability, oil-wet, carbonate reservoir cores. Proceedings of the Abu Dhabi International Petroleum Exhibition and Conference.

[26] Al-Murayri M.T., Kamal D.S., Al-Sabah H.M., AbdulSalam T., Al-Shamali A., Quttainah R., Glushko D., Britton C., Delshad M., Liyanage J. Low-Salinity Polymer Flooding in a High-Temperature Low-Permeability Carbonate Reservoir in West Kuwait. Proceedings of the SPE Kuwait Oil and Gas Show and Conference.

[27] Leon J.M., Castillo A.F., Perez R., Jimenez J.A., Izadi M., Mendez A., Castillo O.P., Londoño F.W., Zapata J.F., Chaparro C.H. A Successful Polymer Flood Pilot at Palogrande-Cebu, A Low Permeability Reservoir in the Upper Magdalena Valley, Colombia. Proceedings of the SPE Improved Oil Recovery Conference.

[28] Musevic I., Skarabot M., Tkalec U., Ravnik M., Zumer S. (2006). Two-Dimensional Nematic Colloidal Crystals Self-Assembled by Topological Defects. Science.

[29] Su X., Cunningham M.F., Jessop P.G. (2013). Switchable viscosity triggered by CO_2_ using smart worm-like micelles. Chem. Commun..

[30] Lin S., Theato P. (2013). CO_2_-responsive polymers. Macromol. Rapid Commun..

[31] Zhang Y., Feng Y., Wang J., He S., Guo Z., Chu Z., Dreiss C.A. (2013). CO_2_-switchable wormlike micelles. Chem. Commun..

[32] Zhang Y., Feng Y., Wang Y., Li X. (2013). CO_2_-Switchable Viscoelastic Fluids Based on a Pseudogemini Surfactant. Langmuir.

[33] Luo X., Zheng P., Gao K., Wei B., Feng Y. (2021). Thermo- and CO_2_-triggered viscosifying of aqueous copolymer solutions for gas channeling control during water-alternating-CO_2_ flooding. Fuel.

[34] Shen H., Yang Z., Li X., Peng Y., Lin M., Zhang J., Dong Z. (2021). CO_2_-responsive agent for restraining gas channeling during CO_2_ flooding in low permeability reservoirs. Fuel.

[35] Wu Y., Liu Q., Liu D., Cao X.P., Yuan B., Zhao M. (2023). CO_2_ responsive expansion hydrogels with programmable swelling for in-depth CO_2_ conformance control in porous media. Fuel.

[36] Zhang D., Li Y., Bao Z. (2005). A laboratory experimental study of feasibility of polymer flood for middle-low permeability reservoirs in Daqing. Oilfield Chem..

[37] Pal N., Zhang X., Ali M., Mandal A., Hoteit H. (2022). Carbon dioxide thickening: A review of technological aspects, advances and challenges for oilfield application. Fuel.

[38] Yang J., Dong H. (2016). CO_2_-responsive aliphatic tertiary amine-modified alginate and its application as a switchable surfactant. Carbohydr. Polym..

[39] Fan W., Tong X., Farnia F., Yu B., Zhao Y. (2017). CO_2_-Responsive Polymer Single-Chain Nanoparticles and Self-Assembly for Gas-Tunable Nanoreactors. Chem. Mater..

[40] Kumar S., Mandal A. (2017). A comprehensive review on chemically enhanced water alternating gas/CO_2_ (CEWAG) injection for enhanced oil recovery. J. Pet. Sci. Eng..

[41] Kumar N., Sampaio M.A., Ojha K., Hoteit H., Mandal A. (2022). Fundamental aspects, mechanisms and emerging possibilities of CO_2_ miscible flooding in enhanced oil recovery: A review. Fuel.

[42] Li H., Zheng S., Yang D. (2013). Enhanced swelling effect and viscosity reduction of solvent (s)/CO_2_/heavy-oil systems. Spe J..

[43] Chaturvedi K.R., Trivedi J., Sharma T. (2019). Evaluation of Polymer-Assisted Carbonated Water Injection in Sandstone Reservoir: Absorption Kinetics, Rheology, and Oil Recovery Results. Energy Fuels.

[44] Zhao J., Torabi F., Yang J. (2020). The synergistic role of silica nanoparticle and anionic surfactant on the static and dynamic CO_2_ foam stability for enhanced heavy oil recovery: An experimental study. Fuel.

[45] Hamidi H., Haddad A.S., Mohammadian E., Rafati R., Azdarpour A., Ghahri P., Ombewa P., Neuert T., Zink A. (2017). Ultrasound-assisted CO_2_ flooding to improve oil recovery. Ultrason. Sonochem..

[46] Yang Y., Li W., Zhou T., Dong Z. (2018). Using Polymer Alternating Gas to Enhance Oil Recovery in Heavy Oil. IOP Conf. Ser. Earth Environ. Sci..

[47] Gandomkar A., Torabi F., Riazi M. (2020). CO_2_ mobility control by small molecule thickeners during secondary and tertiary enhanced oil recovery. Can. J. Chem. Eng..

[48] Manan M.A., Farad S., Piroozian A., Esmail M.J.A. (2015). Effects of Nanoparticle Types on Carbon Dioxide Foam Flooding in Enhanced Oil Recovery. Pet. Sci. Technol..

[49] Zaberi H.A., Lee J.J., Enick R.M., Beckman E.J., Cummings S.D., Dailey C., Vasilache M. (2019). An experimental feasibility study on the use of CO_2_-soluble polyfluoroacrylates for CO_2_ mobility and conformance control applications. J. Pet. Sci. Eng..

